# Use of Telemedicine for Emergency Triage in an Independent Senior Living Community: Mixed Methods Study

**DOI:** 10.2196/23014

**Published:** 2020-12-17

**Authors:** Kelsi Carolan, David C Grabowski, Ateev Mehrotra, Laura A Hatfield

**Affiliations:** 1 School of Social Work University of Connecticut Hartford, CT United States; 2 Department of Health Care Policy Harvard Medical School Boston, MA United States

**Keywords:** telemedicine, telehealth, independent senior living communities, emergency care, first responders

## Abstract

**Background:**

Older, chronically ill individuals in independent living communities are frequently transferred to the emergency department (ED) for acute issues that could be managed in lower-acuity settings. Triage via telemedicine could deter unnecessary ED transfers.

**Objective:**

We examined the effectiveness of a telemedicine intervention for emergency triage in an independent living community.

**Methods:**

In the intervention community, a 950-resident independent senior living community, when a resident called for help, emergency medical technician–trained staff could engage an emergency medicine physician via telemedicine to assist with management and triage. We compared trends in the proportion of calls resulting in transport to the ED (ie, primary outcome) in the intervention community to two control communities. Secondary outcomes were telemedicine use and posttransport disposition. Semistructured focus groups of residents and staff were conducted to examine attitudes toward the intervention. Qualitative data analysis used thematic analysis.

**Results:**

Although the service was offered at no cost to residents, use was low and we found no evidence of fewer ED transfers. The key barrier to program use was resistance from frontline staff members, who did not view telemedicine triage as a valuable tool for emergency response, instead perceiving it as time-consuming and as undermining their independent judgment.

**Conclusions:**

Engagement of, and acceptance by, frontline providers is a key consideration in using telemedicine triage to reduce unnecessary ED transfers.

## Introduction

Nationwide, roughly one million people live in independent senior living communities [1], and residents often receive fragmented medical services. Senior living communities provide many amenities, but residents typically receive medical care from off-site physicians. When residents of senior living communities experience a new medical issue, the lack of an on-site physician may lead to unnecessary transfers to the emergency department (ED). Introducing on-site medical services within independent living communities may safely prevent unnecessary transfers [2,3] but may also be cost-prohibitive.

Telemedicine is a promising, less costly alternative for acute care in community settings [4]. In a randomized study in Massachusetts, United States, the introduction of off-hour telemedicine coverage in nursing homes decreased the hospital transfers by 11% [5]. However, the effectiveness of telemedicine for triage in independent senior living communities has never been evaluated. We studied the introduction of telemedicine for emergency triage at one such community. The goal of the program was to reduce the number of medical calls that resulted in transfers to the ED.

## Methods

### Overview

Telemedicine was introduced at one of three independent senior living communities in California, United States, managed by a single company. The intervention community was home to 950 residents. The remaining two communities, with similar staff models and residents, did not implement the program and served as comparators.

### Clinical Services at Three Senior Living Communities

Safety staff in all three communities provided 24-hour response to resident calls for assistance, typically via a call pendant. Nearly all safety staff were certified as emergency medical technicians (EMTs). Residents of the communities were aware that the safety staff were trained for emergency medicine, as this was one of the selling points of living in such a community. As such, resident calls directly to the emergency telephone number 911 were rare. Upon arrival at a call, safety staff completed an initial assessment and made a triage decision between a life-threatening issue (ie, advanced life support [ALS] transport to the ED), an urgent issue (ie, basic life support [BLS] transport to the ED), less urgent care that still required same-day assessment (ie, transport to the ED via personal vehicle), or treat and release. Treat and release could involve follow-up in the following days with a personal physician. Obviously, residents indicated their wishes and in some cases decided against transfer to the ED against medical advice. For ED transfers, the safety staff member met the ambulance and facilitated the transfer. Following the call, staff documented the call in an electronic call log. Resident calls directly to 911 were not included in this study.

The three communities each had an on-site clinic staffed by nurses and nurses’ aides during weekday hours. The on-site clinic focused primarily on medication delivery or minor issues such as a rash. These staff did not typically become involved in acute complaints.

### Telemedicine Triage Intervention

After the introduction of telemedicine in May 2017, safety staff at the intervention community completed the same initial assessment; they then had the option of offering the resident a telemedicine consult. Safety staff were asked to utilize telemedicine for cases that were not clearly urgent and/or if there was uncertainty as to whether transport was warranted. If the safety staff felt the call was a life-threatening emergency, the telemedicine intervention was not utilized. If telemedicine was offered and the resident accepted, safety staff initialized a videoconference call on a tablet that they brought to all calls.

The telemedicine visits were provided by an emergency medicine physician who worked for a large national emergency medicine staffing company that had introduced the telemedicine service as a new care option. The visits were provided for free as part of this pilot project. There were a number of presentations about the service at regular facility meetings, including one where senior leadership from the telemedicine company attended a town hall meeting to promote awareness of the service and answer questions.

The responding emergency medicine physician worked remotely with the safety staff member to conduct an exam, using observations aided by stethoscope, blood pressure cuff, and pulse oximetry. The safety staff member, physician, and resident decided whether an ED transfer was warranted. The remaining response and follow-up procedures were the same.

### Quantitative Analysis

Our quantitative analysis used deidentified medical call logs from the three communities. Variables included date; chief complaint; whether telemedicine was offered and accepted, if applicable; the outcome; and whether transported residents were admitted to the hospital. We excluded accidental activations and nonmedical calls. The format of the call logs differed among the three communities, requiring some reconciliation (see Multimedia Appendix 1). Our primary outcome was the proportion of calls resulting in residents being transported to the ED. Secondary outcomes were telemedicine use (ie, offers and acceptance) and disposition after transfer to the ED (ie, admitted to hospital or not).

Call log data were available from January 2017 to August 2018. In May 2017, telemedicine was introduced; in December 2017, a new policy required safety staff to offer telemedicine on all calls for which BLS transport would otherwise be called. Previously, this was at the discretion of the staff. Thus, we divided the data into three periods: the *Pre* period was from January to April 2017, the *Early* period was from May to November 2017, and the *Late* period was from December 2017 to August 2018. Quantitative analyses were performed in R v3.5.1 (The R Foundation).

### Qualitative Analysis

#### Overview

Qualitative data can provide key insights into the decision-making processes of subjects [6], rendering it particularly useful for investigating facilitating factors and barriers in the adoption of novel medical interventions. Toward this aim, the authors conducted four semistructured focus group interviews in June 2018.

#### Participant Recruitment and Data Collection

Resident focus groups were advertised during the intervention facility’s regularly held, facility-wide resident town halls and were further advertised through flyers posted throughout the facility. Two resident focus groups were conducted by the authors and included 19 residents with and without personal experience of telemedicine calls (see interview guides in Multimedia Appendix 2). Participating residents were asked to fill out anonymous demographic forms, but very few did so, rendering the demographic information collected insufficiently representative of the sample to report. Staff were recruited through the facility’s resident director, who asked staff both on and off shift to consider participating and compensated staff for their time spent in a focus group. The two staff focus groups included 4 safety staff and 3 nurses’ aides from the on-site clinic. Informed consent was obtained from all participants prior to commencing the focus group interviews and recording. The study was approved by the Institutional Review Board of the Longwood Medical Area in Boston, Massachusetts, United States.

#### Data Analysis

Interviews were recorded and professionally transcribed. The first author led qualitative data analysis, using a thematic analysis approach and NVivo 12 software (QSR International) to organize resulting themes. Employing a thematic analysis approach to analyzing the data allowed for the identification of themes, both within each unit of data analysis (ie, each transcribed focus group) and across the data set [7]. Thematic analysis is a six-step qualitative method of finding and interpreting patterns in the data: familiarization with the data, generating initial codes, searching for themes, reviewing themes, defining and naming themes, and producing a report [7]. The authors utilized a theory-driven approach to thematic analysis, which involves focusing on a previously identified research objective [7]; in the case of this study, the objective was to identify factors that facilitated or acted as barriers to resident participation in the intervention.

The first step of thematic analysis, familiarization with the data, began during data collection and preparation, with the authors discussing and taking notes between focus groups on initial ideas and potential patterns. This process continued through the first author’s verification of the transcripts for accuracy, which involved multiple close readings of each transcript to confirm alignment with the audio recordings. The second step of thematic analysis, initial coding, involves beginning to identify and group data into relevant categories [7]. The first author looked for manifest and latent data, coding for content explicitly stated in the data, as well as attending to statements that highlighted a participant’s attitude or assumptions [7].

After completing initial coding, the first author organized initial codes into related groups and began to search for preliminary themes within each of these lists of codes organized by topic [7]. At this juncture, the first author presented organized initial codes and resulting preliminary themes to the senior author for feedback, with the two authors discussing these preliminary results until a consensus on preliminary themes was reached. Next, qualitative transcripts were loaded into NVivo 12, and the first author utilized NVivo 12 to organize and review the initial themes identified through this consensus process. This review involved reconsidering both the relevance and importance of each initial theme as it related to the research objective and the congruence of identified themes across the data set as a whole, resulting in a further refined set of themes that were then named and defined [7]. The analysis process concluded with the first author presenting identified themes to all four authors, engaging in additional discussion and refinement of themes until consensus was reached. As all four authors participated in the data collection via co-leading focus groups, engaging in this final step supported the trustworthiness of findings by creating an opportunity to re-examine the extent to which the presented themes sufficiently represented the data and to build consensus on the salience of finalized themes.

## Results

### Quantitative Results

**Figure 1:**
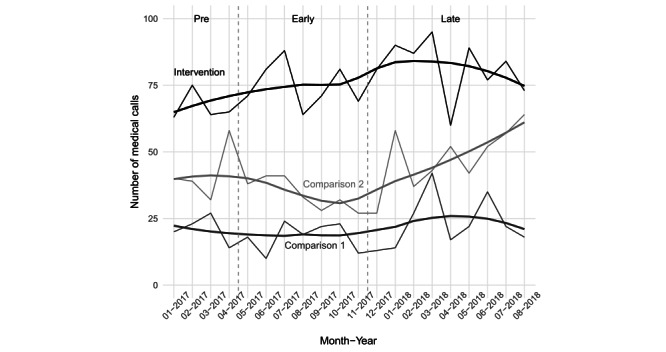
Number of medical calls at the intervention and the two comparison communities. Dashed vertical lines indicate the demarcations between *Pre* and *Early* periods (ie, the start of telemedicine in May 2017) and between *Early* and *Late* periods (ie, the start of the new telemedicine use policy in December 2017).

**Figure 2:**
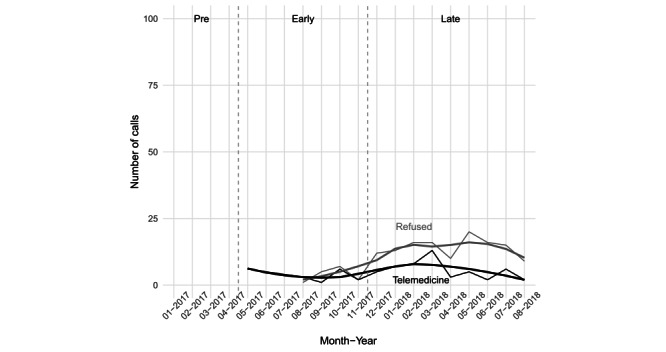
Number of telemedicine calls and refusals at the intervention community. Dashed vertical lines indicate the demarcations between *Pre* and *Early* (ie, the start of telemedicine in May 2017) and between *Early* and *Late* periods (ie, the start of the new telemedicine use policy in December 2017).

Average monthly call volumes rose over time in all three communities: from 67 (SD 6) to 80 (SD 11) in the intervention community, and from 42 (SD 11) to 46 (SD 13) and from 21 (SD 5) to 23 (SD 10) in the two comparison communities, respectively (see Figure 1). The average number of telemedicine calls per month was 4.2 (SD 1.9) in the *Early* period (5.5% of all calls, 25/456) and 5.3 (SD 3.5) in the *Late* period (6.6% of all calls, 53/805) (see Figure 2). The policy to mandate use of telemedicine for BLS calls increased the rate of refusals from 2.9% (13/456) to 16.0% (129/805) of calls.

**Figure 3:**
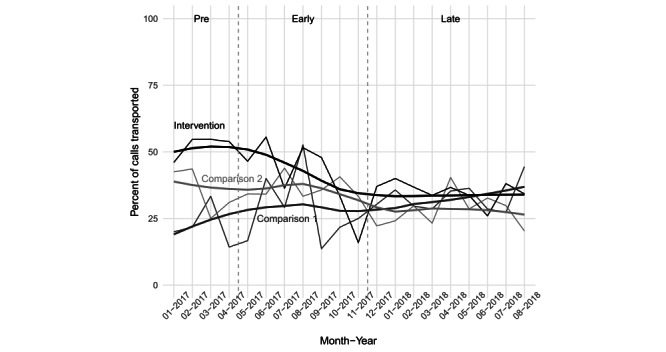
Percentage of calls resulting in transport to the emergency department by ambulance at the intervention and two comparison communities. Dashed vertical lines indicate the demarcations between *Pre* and *Early* (ie, the start of telemedicine in May 2017) and between *Early* and *Late* (ie, the start of the new telemedicine use policy in December 2017).

At the intervention community, the fraction of calls resulting in ED transfer decreased from 52.4% (140/267) in the *Pre* period to 33.5% (270/805) in the *Late* period (ie, 18% decrease) (see Figure 3). At the comparison communities, calls decreased by 8% in one and increased by 8% in the other. To assess whether the change observed in the intervention community could be driven by growth of telemedicine, we compared the observed change in the ED transport rate at the intervention community to the theoretical change, in which every telemedicine call that did not result in ED transport had instead resulted in transport. This yields an upper bound on the possible impact of telemedicine. Of the 53 total telemedicine calls in the *Late* period, 37 (70%) were not transported. If, in the absence of telemedicine, all of these had been transported, the maximum possible effect of telemedicine would have been to decrease the transport rate by 5% versus by the observed rate of 18%.

**Figure 4:**
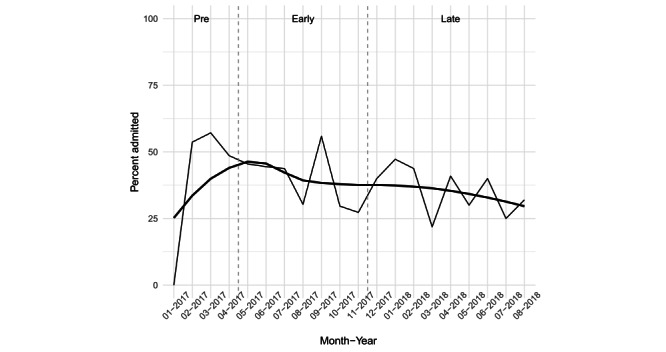
Percentage of transported calls from the intervention community that resulted in a hospital admission. Dashed vertical lines indicate the demarcations between *Pre* and *Early* (ie, the start of telemedicine in May 2017) and between *Early* and *Late* periods (ie, the start of the new telemedicine use policy in December 2017).

If telemedicine prevents unnecessary ED transfers, the remaining transfers likely would be higher acuity and more likely to result in hospital admissions; however, we saw the opposite (see Figure 4). Over time, the percentage of transported calls that were admitted decreased from 42.1% (59/140) in the *Pre* period and 42.2% (86/204) in the *Early* period to 35.2% (95/270) in the *Late* period. Therefore, our evidence is insufficient to attribute the decrease in the transport rate at the intervention community to telemedicine.

### Qualitative Results: Staff Perspectives

#### Overview

EMT-trained safety staff did not perceive telemedicine as a valuable tool, viewing it as undermining their autonomy in decision making and increasing their workload. Reducing resident transports to the ED was not a goal embraced by safety staff, who described approaching all calls with the assumption that transport would likely be necessary.

#### Telemedicine: “Delaying the Inevitable”

Safety staff approached calls with the perspective that resident transport to the ED was likely: “We normally respond to all emergencies like probably they are going to consist of transports.” Another safety staff participant reiterated, “...the majority of the calls that I get, they usually warrant transport anyway, so I just go, ‘There’s no point [in a telemedicine consult].’ That’s kind of where I’m at with it.” If the medical need for transport appeared uncertain, staff preferred to call an ambulance: “If there is a borderline [case]...maybe you err on the side of just calling EMS [emergency medical services] out to handle that assessment.” Operating from the perspective that transport was likely necessary and/or best in the case of uncertainty, staff viewed telemedicine as an unnecessary obstacle, delaying the resident’s needed emergency transport: “...we have to wait for the telemedicine conference, it still ends up in transport anyway, so it’s just delaying the inevitable.” Thus, safety staff also perceived telemedicine as undermining their professional judgment:

That was my first [time using telemedicine] and she ended up going out anyway. The resident was...complaining of dizziness, couldn’t make it back to her unit from outside. The [telemedicine] doc was basically like, “Oh, just go rest. Check on her every 20 minutes.” Went and checked on her 20 minutes later. She had nausea and vomiting and was passed out on the floor, couldn’t keep herself up, so she still went out on ALS transport. That was one of them that...I was definitely frustrated, because I already thought she should go out ALS...That was, for me, I never really used it after that. I used it a couple of times, and each time it still ended up in the transport, too.

This safety staff participant independently determined that transport was necessary, and regarded this decision as undermined by the telemedicine physician. When transport did end up being necessary in this case, the safety staff participant’s view of telemedicine consult as an obstacle rather than an aide was cemented.

#### Telemedicine Is Not a Good “Fit”

Staff expressed that telemedicine was of limited use for the community’s residents: “...the majority of stuff we deal with here, it doesn’t fit the bill. Old people don’t bounce. They fall and break.” Another participant described common conditions for which residents call safety staff: “...shortness of breath, and fevers...but most of the time, they’re 102, they’re septic, they’re coughing up phlegm, their lungs are nasty. You’re like, ‘They need to go.’” One participant indicated that falls with pain are frequent: “I always tell them, ‘I can’t see through you, so you’re going to have to go get an x-ray or an MRI [magnetic resonance imaging] or whatever it needs to be.’” Staff highlighted that they were accustomed to making independent triage decisions and that the telemedicine physician “asking the same questions” was not helpful.

#### Telemedicine for Nonemergency Use

Safety staff felt that the program’s goal to deter ED visits was the wrong goal and that telemedicine would better address minor medical concerns at the on-site clinic. Safety staff indicated that “the resistance you have from our department is we’re emergency response,” whose “primary goal is security, medical response.” The nursing staff at the on-site clinic “deal with the nonemergency, ‘Hey, I don’t feel well,’ and they walk into their office looking for the Tylenol.” Safety staff indicated more willingness to utilize the telemedicine consult if the safety staff called to the case had already determined for themselves that transport was not necessary. A participant indicated that telemedicine could be beneficial if and when “I don’t think it warrants going to the hospital. It may be an alternative to going to their own doctor or to an urgent care. If I don’t see emergency treatment as really necessary.” The safety staff’s self-description of their role as emergency responders and the emphasis on telemedicine as potentially more suitable for nonemergency cases highlights their perception of telemedicine intervention as inappropriate for emergency use.

#### Perceived Burden Without Perceived Benefit

Safety staff indicated that telemedicine increased their workload, describing the software as difficult to navigate: “Just the sheer amount of time that it takes...typically there’s three [safety staff on duty]. We have well over 900 residents we’re checking on. Sometimes the telemedicine can take a little longer than I think is warranted.” This was echoed by another staff member who described telemedicine as increasing the difficulty of juggling multiple demands: “It can be time-consuming. We have a lot of things going on, more than one medical going on...it can be very time-consuming.” Safety staff participants also described a number of technical issues that acted as a barrier to use, such as inconsistent wireless internet connectivity throughout the facility: “...that’s come up in certain parts of the campus...We just try and make do and move around if possible.” In combination with the safety staff’s perception of telemedicine as inappropriate for emergency situations, the staff’s experience of telemedicine as additional work led to the perception of telemedicine as adding burden without adding value.

#### Residents Are Reluctant

Safety staff also reported that residents were reluctant to use telemedicine. One safety staff member said, “Honestly, they don’t directly request it, and I think a lot of that has to do with they don’t want to change from the old-school days of actually seeing a doctor. A lot of them, they go, ‘No, they can’t do anything for me over the phone or over the tablet. I actually need to go in.’” Another staff member agreed, saying, “...when I do offer it to residents, they only want to see their doctor and then they want to be in person...to talk to someone who knows them personally.” One participant described this perceived reluctance as a lack of comfort with receiving medical care through an unfamiliar medium:

I think it’s a generational thing. I think once all of us are old, you hand me a tablet and go “Here’s the doctor,” I’m going to go, “Alright, let’s do it,” but...It was different back when they saw a doctor, they had that personal care, so a lot of them, I think that’s part of the problem. They’re like, “I just want to see my doctor.”

### Qualitative Results: Residents

#### Overview

In contrast to staff perceptions, resident focus group participants described multiple benefits of using telemedicine. Residents emphasized that avoiding an ambulance transport and having their concerns successfully resolved by the telemedicine physician were the most significant benefits of a telemedicine consult.

#### Avoiding the ED

Residents expressed a strong interest in avoiding trips to the ED whenever possible, mentioning the long wait times, financial costs, and potential health risks of ED visits. Residents interviewed identified avoiding an ED visit as a primary benefit of using the telemedicine intervention. A resident described satisfaction with the quality of the telemedicine consults she received, emphasizing that an unnecessary transfer to the ED was prevented in both of her experiences with the telemedicine intervention:

The doctor I spoke with was very understanding and he went through all the questions he should pertaining to my problem, and I was very reassured...he had my medical record right in front of him, and so he knew all of my problems and that also enlightened him as to how to treat me. The second time it was the same experience and the same thing. I had another doctor, and I was very pleased. In both instances it did save a trip going to the ER.

Another resident emphasized the value of telemedicine in preventing emergency transport:

Well, I had this situation before, so I thought, “Okay, if this keeps me out of the hospital, good.” And he did. He stayed on with me for a long time and after he left, everything was fine and I didn’t need to go to the hospital...I think that’s where telemedicine is a good thing, saving people going to the emergency room.

Other residents who had not yet tried telemedicine indicated a willingness to do so if the opportunity arose. A resident expressed the following:

I think it’s a fine service that would be very beneficial and, in many cases, prevent someone from going to an emergency room if they didn’t need to. I’d rather have a medical opinion available to me on the spot to make that determination... I would not have the EMTs say, “Well, we’re going to take you...” So I think it’s a good deal.

#### Reinforcing the Need for Emergency Transport

Residents also described an unanticipated benefit of telemedicine: encouraging hesitant residents to go to the emergency room when necessary. A resident described his experience as follows:

The skeptical patient is the one that this is directed to. I passed out after walking up a hill, and when I woke up I felt good...So I just thought, “Well, it’s just passing out. I’m going to go ahead...” And then the telemedicine person said, “Well, assuming your recent history in hospitals, we would recommend that you go to the emergency room.”

The participant described how he agreed to the transport and was diagnosed with a “fatal heart condition” in the ED. He concluded, “Everybody I’ve talked to about telemedicine, I just tell them they saved my life...convincing me that it was necessary to go. I wouldn’t have gone.”

#### Resident Concerns: Delaying Treatment

Resident focus group data indicated that residents were satisfied with the results of telemedicine use and/or expressed a willingness to try it. A few residents did indicate concern that telemedicine would delay treatment:

My concern is it delays my journey to a physician. And sometimes you need a CT [computed tomography] scan, sometimes you need some really heavy meds, and if I have to spend 40 minutes at home going through telemed...that just slows the whole process down. So, to me, it’s a negative...If you’re in a lot of pain, you don’t want to mess with telemedicine. You want to get somewhere where you can be treated right away.

However, the majority of residents expressed confidence in their ability to determine when telemedicine might be useful.

## Discussion

### Principal Findings

Unnecessary ED visits are a major source of excess morbidity and spending [8] and are common in independent senior living communities. Telemedicine has the potential to help deter unnecessary ED visits [4]. In our evaluation of a telemedicine option in a senior living community, we did not observe a clear decrease in ED transfers due to the telemedicine intervention. The use of the tool was low, which appeared to be driven by staff opposition to the model. Residents viewed the service more favorably, with several asserting it prevented an unnecessary transfer.

Our findings echo other studies in this area where securing provider buy-in is an ongoing challenge for many organizations implementing telemedicine interventions [9]. In a study of a telemedicine intervention in several skilled nursing facilities, some nurses refused to use the service [5]. In evaluations of heart failure telemedicine interventions, medical personnel felt it reduced autonomy and increased workload [10]. The views of frontline providers are key to successful implementation of telemedicine interventions.

Minimal research has explored the feasibility and acceptability of telemedicine interventions involving first responders as frontline providers [11]. The intervention facility’s senior management considered EMT-trained safety staff as an advantage, as staff would be able to effectively assist the remote physician. However, the safety staff viewed the intervention as undermining their autonomy. Traditionally, prehospital first responders are trained to provide minimal treatment to stabilize patients for emergency transport, with successful transfer to the ED as the goal [12]. EMTs embracing reduced ED transport as a goal would require a significant shift in professional mentality and culture.

Staff may have benefitted from further education on identifying potentially avoidable transfers, the harm to residents of unnecessary transfers, and the opportunity to work and learn in cooperation with remote physicians. Designating an individual frontline provider as a *champion* for the program may help to encourage telemedicine adoption by other frontline staff [9,13]. In this case, the champion was the director of the safety staff, who was not a frontline provider. Having a frontline provider as champion may have helped communicate staff concerns and helped educate the safety staff about the program.

Financing of telemedicine interventions in independent living settings presents an additional key challenge. This program was offered free of charge to residents living in the independent living community because the services were donated in kind by the telemedicine provider as part of the demonstration project. For telemedicine programs to be more widely adopted, they will need to be financed by residents or third-party payers. Payers like Medicare Advantage or accountable care organizations that are at risk for transfers to the ED appear willing to invest in these programs. There have already been clinical investments in nursing homes [14] and assisted living facilities [3,15] by Medicare Advantage plans. The Centers for Medicare and Medicaid Services recently released a new payment model reimbursing emergency ambulance service providers for telemedicine interventions provided to Medicare beneficiaries [16]. This may incentivize the increased use of telemedicine by ambulance service providers and increase interest in the adoption of telemedicine services in long-term care settings.

### Limitations

Our study has several key limitations. It is based on the adoption of telemedicine at one senior living community, which may not generalize to other communities. The focus groups were convenience samples that may not be representative of the entire population of residents and staff. Residents of these communities, on average, were from high socioeconomic strata and are not representative of the general population of older adults in independent senior living communities. The two comparison communities used slightly different staffing models and call log data formats, limiting the comparability of the data. The number of telemedicine calls each month was very low, limiting statistical power. This study was conducted prior to the COVID-19 pandemic, during which the use of telemedicine has grown rapidly. A telemedicine triage intervention may now be more attractive to both frontline providers and residents.

### Conclusions

We found that the introduction of telemedicine in an independent living community did not clearly reduce transfers to the ED. The intervention had low uptake due to limited buy-in by frontline staff. Future interventions involving telemedicine use in independent living communities should carefully consider how best to prepare and engage frontline providers.

## Appendix

Multimedia Appendix 1 Details of the call log variables.

Multimedia Appendix 2 Semistructured interview guides.

## Abbreviations

ALS: advanced life support
BLS: basic life support
CT: computed tomography
ED: emergency department
EMS: emergency medical services
EMT: emergency medical technician
MRI: magnetic resonance imaging
