# Supplementary material for: Use of Telemedicine for Emergency Triage in an Independent Senior Living Community: Mixed Methods Study
Source: J Med Internet Res. 2020 Dec 17;22(12):e23014. doi: 10.2196/23014 (PMC7775198; doi:10.2196/23014)
Supplement: Multimedia Appendix 1 [file jmir_v22i12e23014_app1.doc]

# Appendix 1: Details of the call log variables

|  | Intervention | Comparison 1 | Comparison 2 |
| --- | --- | --- | --- |
| Date | [Consistently reported] | [Consistently reported] | [Consistently reported] |
| Fire alarm | -- | Yes or blank | -- |
| Property damage | -- | Yes or blank | -- |
| Resident  needing assistance | -- | Free text | -- |
| Smoke alarm | -- | Yes or blank | -- |
| Suspicious activity | -- | Yes or blank | -- |
| Theft/missing item | -- | Yes or blank | -- |
| 911 Call | -- | Yes or blank | -- |
| Bath alarm | -- | Yes or blank | -- |
| Pendant alarm | -- | Yes or blank | -- |
| Death of resident | -- | Yes or blank | -- |
| Missing resident | -- | Yes or blank | -- |
| Resident confused | -- | Yes or blank | -- |
| Resident fall | -- | Yes or blank | -- |
| Resident illness | -- | Yes or blank | -- |
| Resident injury | -- | Yes or blank | -- |
| Accidental/malfunction | -- | Yes or blank | 1 or blank |
| Chief complaint | Free text | -- | Free text |
| Incident | Fall,  Medical,  CPR/AED,  Trauma-other | -- | -- |
| Telemedicine | Yes (pre-consent),  Yes (post-consent),  No, Refused, N/A | -- | -- |
| Transported | Advanced life support, basic life support,  non-transport,  private vehicle,  against medical advice, urgent care | Yes or blank | -- |
| Post-transport disposition | Returned,  admitted,  N/A | -- | -- |
| Basic life support transport | -- | -- | 1 or blank |
| Advanced life support transport | -- | -- | 1 or blank |
| Non-transport | -- | -- | 1 or blank |
| Against medical advice | -- | -- | 1 or blank |
