# Supplementary material for: Use of Telemedicine for Emergency Triage in an Independent Senior Living Community: Mixed Methods Study
Source: J Med Internet Res. 2020 Dec 17;22(12):e23014. doi: 10.2196/23014 (PMC7775198; doi:10.2196/23014)
Supplement: Multimedia Appendix 2 [file jmir_v22i12e23014_app2.doc]

# Appendix 2: Semi-Structured Interview Guides

## Semi-structured Interview Guide: Resident Focus Groups

Introductory Script: We have asked you to join us today because we want to understand your experiences when you call for assistance. Your perspective is important to us. To help get us started, I’m going to read you an example of a health situation someone might experience. As you listen, please think about what you might do if you were in this situation.

Case/vignette: Mary lives at a senior living community. One night when she got up to use the bathroom in the night, she slipped and fell. She was able to get up and get to her couch, but when she stood up, her right ankle hurt badly. Concerned, she pressed her call button for help. George, a member of the safety staff, arrived a few minutes later. After George checked out Mary’s ankle, which continued to hurt, he discussed Mary’s options with her. George said that he could call an ambulance to take Mary to the nearest emergency room, or with his assistance, Mary could speak with an emergency medicine doctor via videoconference on a tablet. Mary could also decline both of these options.

Vignette Follow-up questions:

What do you think Mary should do? What seems like the best option?

- What would you do in Mary’s situation? What would you advise Mary to do if she were your spouse or friend?
- What concerns might Mary have about talking to the emergency medicine doctor via videoconference?
- What might be the benefits of talking to the emergency medicine doctor via videoconference?
- What concerns might Mary have about going to the emergency room?
- What might be the benefits of going to the emergency room?
- If Mary had a different type of injury or illness, would you give her different advice?
- Are there some situations in which Mary might prefer talking to the emergency medicine doctor via videoconference, and some where she might prefer going directly to the emergency room?
- How does Mary’s personal doctor play a role in this decision? Should she wait to see him/her?

Personalized follow-up questions:

We asked each of you to join us here today because you may have found yourself in a similar situation to Mary in the last few months. If the safety staff felt that you did not need to go to the ER right away, they may have offered you the option of going straight to the emergency room or speaking with an emergency medicine doctor via videoconference.

Can you tell us about the decision you made, and why you chose that option?

- What made that feel like the best decision for you?
- What stopped you from wanting to [choose the other option]?
- What did you find frustrating or concerning about [relevant option]?
- What were the benefits of [chosen option]?
- If you had to call for assistance again for a different injury or illness, what situations would you prefer [choose the other option]?

Suggestions for improvement

- Were your options explained clearly? Was there anything unclear, confusing or concerning about the way the [telemedicine intervention] was explained to you?
- Had you heard about the [telemedicine intervention] prior to your call for assistance? How did you hear about it? (If telemedicine intervention was chosen: Was it similar or different to what you expected?)
- What else could be done to make the [telemedicine intervention] easier to use? Easier to understand? What changes or improvements would you suggest?

## Staff focus groups/interviews, semi-structured interview guide

Introductory Script: We have asked you to join us today because we are interested in learning more about what happens when you respond to an emergency call from one of the residents here. We appreciate you taking the time to be here, as your specific perspectives are valuable to us.

Understanding the emergency protocol:

To begin with, can you walk us through your emergency response protocol step-by-step, so that we can better understand it from your perspective?

Prompts:

- When a resident [presses their emergency response button], what is the first thing that happens? What is the first step that you as a staff member take?
- What happens when you get to the resident’s room, what are the first few things you do?
- If you offer the resident the telemedicine intervention, how do you explain it to them?
- If they say yes to the telemedicine intervention, what are the steps involved?
- If they say no to the telemedicine intervention, what you do next?

Examples of actual emergency calls:

- Tell us about a recent emergency call you responded to
- Tell us about a recent emergency call you responded to, and the resident said yes to the telemedicine intervention
- Tell us about a recent emergency call when the resident said no to the telemedicine intervention
- Tell us about a time that telemedicine intervention went well/didn’t go well

Changes to Protocol:

- How are these responses/calls different, now that you are offering the telemedicine intervention to some residents?

Feedback on Intervention:

- What is challenging or frustrating about this new protocol? What concerns have you had? What isn’t working well?
- What are the barriers to offering residents the telemedicine intervention? What concerns do you have about offering it?
- What tends to get in the way of actually carrying out this intervention?
- Is there anything you like about being able to offer the telemedicine intervention? What is going well about this new protocol?
- What do you think could be done to improve the telemedicine intervention? What would you like to see change?
- Are there any other aspects of the emergency response protocol that could be improved?

Resident perspectives:

- Do you have a sense of what residents think about this telemedicine intervention?
- What do you think gets in the way of residents saying yes to the telemedicine intervention?
- What concerns do you think they have?
- Is there anything that might help address these concerns?

Wrap up: Is there anything else you would like to tell us about your experiences with this intervention? Anything else we should know?
